# Supplementary material for: Measuring SARS-CoV-2 neutralizing antibody activity using pseudotyped and chimeric viruses
Source: J Exp Med. 2020 Jul 21;217(11):e20201181. doi: 10.1084/jem.20201181 (PMC7372514; doi:10.1084/jem.20201181)
Supplement: Table S1 — shows Spearman’s correlation statistic for plasma NT50 and mAb IC50 determined using each neutralization assay. [file JEM_20201181_TableS1.docx]

Table S1. Spearman’s correlation statistic for plasma NT_50_ and mAb IC_50_ determined using each neutralization assay

|  | VSV pseudotype | | VSV/SARS-CoV-2 | | SARS-CoV-2 | |
| --- | --- | --- | --- | --- | --- | --- |
|  | Plasma | mAb | Plasma | mAb | Plasma | mAb |
| HIV-1 pseudotype | 0.71 | 0.86 | 0.93 | 0.86 | 0.94 | 0.91 |
| VSV pseudotype |  |  | 0.91 | 0.81 | 0.86 | 0.92 |
| VSV/SARS-CoV-2 |  |  |  |  | 0.89 | 0.93 |
