# Supplementary material for: Measuring SARS-CoV-2 neutralizing antibody activity using pseudotyped and chimeric viruses
Source: J Exp Med. 2020 Jul 21;217(11):e20201181. doi: 10.1084/jem.20201181 (PMC7372514; doi:10.1084/jem.20201181)
Supplement: Table S2 — shows the ratio of plasma NT50 and mAb IC50 for each surrogate assay versus SARS-CoV-2. [file JEM_20201181_TableS2.docx]

Table S2. Ratio of plasma NT_50_ and mAb IC_50_ for each surrogate assay versus SARS-CoV-2

|  | Mean plasma NT_50_ ratio | Mean mAb IC_50_ ratio |
| --- | --- | --- |
| HIV-1 pseudotype | 0.44 ± 0.22 | 1.2 ± 1.6 |
| VSV pseudotype | 0.47 ± 0.45 | 5.8 ± 4.2 |
| VSV/SARS-CoV-2 | 4.6 ± 3.5 | 1.2 ± 0.62 |
